# Alterations in Stem Cell Populations in IGF-1 Deficient Pediatric Patients Subjected to Mecasermin (Increlex) Treatment

**DOI:** 10.1007/s12015-022-10457-2

**Published:** 2022-10-21

**Authors:** Grubczak Kamil, Stożek Karolina, Starosz Aleksandra, Bossowski Filip, Pasławska Marta, Bossowski Artur, Moniuszko Marcin

**Affiliations:** 1 Department of Regenerative Medicine and Immune Regulation, Medical University of Bialystok, Jerzego Waszyngtona 13, 15-269 Bialystok, Poland; 2 Department of Pediatrics, Endocrinology and Diabetes With a Cardiology Unit, Medical University of Bialystok, Jerzego Waszyngtona 17, 15-275 Bialystok, Poland; 3 Department of Allergology and Internal Medicine, Medical University of Bialystok, Marii Sklodowskiej-Curie 24A, 15-276 Bialystok, Poland

**Keywords:** IGF-1 deficiency syndrome, VSEL, HSC, SDF-1

## Abstract

Pathway involving insulin-like growth factor 1 (IGF-1) plays significant role in growth and development. Crucial role of IGF-1 was discovered inter alia through studies involving deficient patients with short stature, including Laron syndrome individuals. Noteworthy, despite disturbances in proper growth, elevated values for selected stem cell populations were found in IGF-1 deficient patients. Therefore, here we focused on investigating role of these cells—very small embryonic-like (VSEL) and hematopoietic stem cells (HSC), in the pathology. For the first time we performed long-term observation of these populations in response to rhIGF-1 (mecasermin) therapy. Enrolled pediatric subjects with IGF-1 deficiency syndrome were monitored for 4–5 years of rhIGF-1 treatment. Selected stem cells were analyzed in peripheral blood flow cytometrically, together with chemoattractant SDF-1 using immunoenzymatic method. Patients’ data were collected for correlation of experimental results with clinical outcome. IGF-1 deficient patients were found to demonstrate initially higher levels of VSEL and HSC compared to healthy controls, with their gradual decrease in response to therapy. These changes were significantly associated with SDF-1 plasma levels. Correlations of VSEL and HSC were also reported in reference to growth-related parameters, and IGF-1 and IGFBP3 values. Noteworthy, rhIGF-1 was shown to efficiently induce development of Laron patients achieving at least proper rate of growth (compared to healthy group) in 80% of subjects. In conclusion, here we provided novel insight into stem cells participation in IGF-1 deficiency in patients. Thus, we demonstrated basis for future studies in context of stem cells and IGF-1 role in growth disturbances.

**Graphical abstract:**

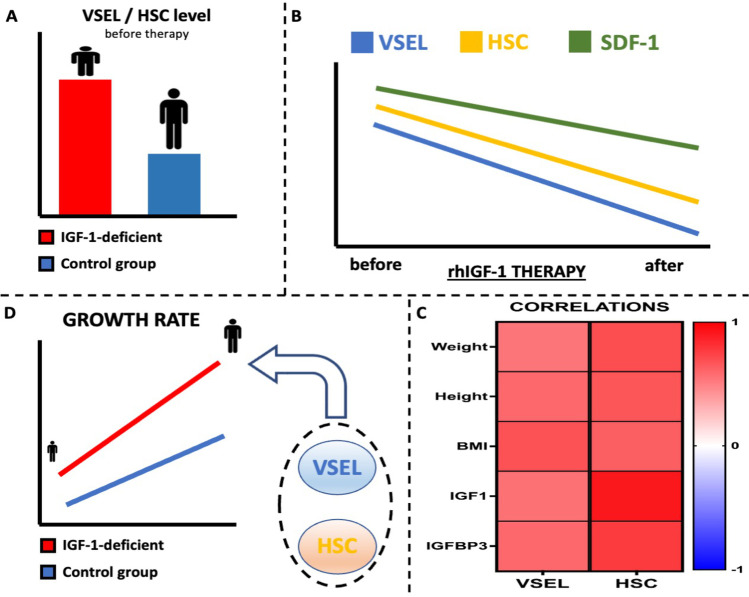

**Supplementary Information:**

The online version contains supplementary material available at 10.1007/s12015-022-10457-2.

## Introduction

Insulin-like growth factor 1 (IGF-1) is a part of essential axis responsible for development and growth of cells and tissues. Due to high homology both, IGF-1 and insulin activity, are related to control of metabolic phenomenon but also longevity [1]. To date, role of IGF-1 signaling pathway was found to be critical for numerous processes associated with growth, including inter alia osteoblasts expansion or nesting of hematopoietic stem cells (HSCs) in general. [2] Noteworthy, IGF-1 proper distribution, function and activity is closely related to its plasma binding protein – IGF-1 binding protein 2 (IGFBP2) [3]. IGF-1 deficiency demonstrates very low prevalence among pediatric patients diagnosed with short stature, constituting approximately 1.2% [4]. Pathological conditions like dwarfism, including Laron syndrome with substantially low levels of IGF-1, allowed us to discover crucial role of that protein in growth and maturation reduction [5]. Laron syndrome is associated with IGF-1 deficiency and mutations in growth hormone (GH) receptor. Due to disabled ability of the receptors to respond to GH, treatment of the IGF-1-deficient patients is based generally on recombinant protein application – mecasermin (available in drug Increlex) [6, 7]. Current studies suggested that despite favorable effects of rhIGF-1 (recombinant human IGF-1), normal values for height are not commonly achieved. Therefore, novel therapeutic approaches are suggested including combination of rhIGH-1 and rhGH or even post-GH receptor agonists (still to be identified) [8, 9].

Significance of VSELs discovery was not only based on the enormous differentiation potential of these cells and possible implementation in regeneration. Most importantly, these cells with embryonic-like characteristic were found to be present in body through whole life in quiescent state [10]. To date, that population of stem cells was found inter alia within cardiac cells [11] and lung tissue [12], apart from their confirmation in bone marrow and peripheral blood [13]. Regarding IGF-1, VSELs were shown to possess its receptor activity through involvement of the related signaling pathways and expression of *IGFR1* gene [14], thus, enabling them to respond to the hormone. However, it is worth to note that these stem cells are able to modify their responsiveness to IGF-1 or insulin through corresponding receptors, and preserve quiescent state [15]. Besides reported role of VSELs in regeneration of tissues damage, elevated levels of these stem cells were also reported in condition associated with development disturbances, like Laron syndrome described above [16]. In contrast to hematopoietic stem cells name, that population was found to give rise not only to leukocytes or other cells of hematopoiesis [17]. More recent data demonstrated that adipocytes can be generated out of HSC cells [18, 19].

In recent years researchers gradually focused more on practical assessment of VSELs role in growth and regeneration of various tissues. Those included inter alia bone defects where VSELs supported other populations of stem cells (present within fraction of bone marrow-derived mononuclear cells—BM-MNC) in osteogenesis and effective reconstruction of the damaged areas [20]. Besides structural tissue losses, VSELs role was suggested to affect limitation of damage progression and maintenance of beta cells proper function in the course of type 1 diabetes [21]. When discussing possible applications of VSELs it is worth to remember that presence of these cells was shown to be closely associated with age, with highest numbers reported in children predominantly [11]. Numerous conditions and therapeutic protocols were demonstrated to be associated with increased levels of VSELs. These include inter alia FSH therapy in women prepared for in vitro fertilization [22].

Apart from VSEL and HSC populations, crucial role of endothelial progenitor cells has been shown in recent years in context of numerous pathological conditions. These include for example metabolic disorders [23]. To date, there are no reports on potential role of these subsets of cells in the course of Laron syndrome. However, considering endothelial progenitor cells (EPC) essential participation in vasculogenesis and angiogenesis [24, 25], their involvement is highly possible in processes accompanying intensive growth of the body.

Here we focused on evaluating changes in circulating stem cells, including VSELs, HSCs and EPCs, in IGF-1-deficient pediatric patients subjected to therapy with mecasermin (Increlex). Our results are the first to demonstrate long-term effects of IGF-1 deficiency syndrome patients’ treatment in context of stem cells. Moreover, we have managed to reveal essential associations between studied cells and growth-related parameters, and therapy influence on these properties.

## Materials and Methods

### Patients and Material

Patients with reported IGF-1 deficiency were enrolled in the study, with age range of 6–12 years. Written consent has been obtained from each patient or legal guardian after full explanation of the purpose and nature of all procedures used. Complete patients’ characteristics was included within supplementary materials (Supp. Figure 1) [26]. In addition, 36 subjects were selected for control group (with proper growth rate, and inflammatory/endocrine/oncological disturbances excluded), with age-matched subjects used for comparisons at before (8.5 ± 2.5 versus 9.7 ± 1.1 years in IGF-1-deficient group) and at the 4-5^th^ year (13.3 ± 2.5 versus 12.8 ± 2.5 years in IGF-1-deficient group) of the therapy implementation. Diagnosis of Primary Insulin-like Growth Factor Deficiency (PIGFD) was based on short stature or growth failure, proper growth hormone production and insufficient production of IGF-1. Other conditions including chronic diseases or poor nutrition has been excluded. Patients were treated with Increlex (mecasermin) – recombinant IGF-1, at initial dosage of 0.04 mg/kg, injected subcutaneously twice a day. Increlex dose was gradually increased by 0.04 mg/kg up to maximum of 0.12 mg/kg. The study protocol was approved by the local Ethical Committee at the Medical University of Bialystok (APK.002.78.2021).

Peripheral blood was collected by venipuncture prior to Increlex application and at control visits in the course of therapy, up to 4–5 years. Whole blood (600 μl) was subjected to immunostaining and flow cytometric analysis, and remaining material was used to obtain plasma. Collected plasma was stored in -80 °C for later immunoenzymatic tests.

### Flow Cytometric Evaluation of Stem Cell Populations

Peripheral blood of IGF-1-deficient patients and healthy controls was subjected to immunostaining with fluorochrome-conjugated monoclonal antibodies: Lin1 FITC (CD3 FITC (clone SK7), CD14 FITC (clone MϕP9), CD16 FITC (clone 3G8), CD19 FITC (clone SJ25C1), CD20 FITC (clone L27), CD56 FITC (clone NCAM16.2)); and others: CD34 FITC (clone 581), CD45 PE (clone HI30), CD133 APC (clone W6B3C1), CD144 PE (clone 55-7H1), CD235a FITC (clone HIR2), CD309 PE (clone 89106) (BD Bioscience) (Supp. Figure 2). Samples were incubated for 25 min, at room temperature, in dark. Subsequently, red blood cells were lysed using 3 ml of Lysing Solution 2 (BD Bioscience) for 10 min, followed by centrifugation at 400 g, 10 min. Cell pellet was disrupted and spinned down one more time in 3 ml of phosphatate-buffered saline (PBS with no calcium and magnesium, Corning), 400 g, 5 min. Stained cells were fixed with 50 μl of CellFix (BD Bioscience) and stored in 4 °C prior acquisition, performed within 8 h. Flow cytometric data were obtained on FACS Calibur flow cytometer (BD Bioscience), and analyzed using FlowJo software (TreeStar Inc., SA, USA).

Complete gating strategy implemented for delineation of VSEL, HSC, and endothelial stem cells was comprehensively described in supplementary materials (Supp. Figure 3). Populations of VSEL and HSC were initially gated from small-sized cells of 2-6 μm (based on the size beads of 1, 2, 4 and 6 μl) (Life Technologies), including strict morphological properties based on the relative size (forward scatter, FSC) and shape/granularity (side scatter, SSC). Subsequently, two-way gating strategy was implemented for mutual control and validation of the rare events analysis results. Thus, one way, population of mature cells in blood was excluded with Lineage1 coctail with addition of anti-CD235a for exclusion of the remaining erythrocytes. Then, cells of interest were distinguished on the basis of progenitor cell marker – CD133, and differential expression of anti-CD45: Lineage-/CD45 + CD133 + (HSC) and Lineage-/CD45-CD133 + (VSEL). The other gating approach first focused on detection of Lineage-negative cells with concomitant expression of CD133, and then, VSEL (Lineage-CD133 + /CD45-) and HSC (Lineage-CD133 + /CD45 +) were gated on the basis of CD45 marker presence. In reference to endothelial stem cells, cells of interest were initially gated out from PBMC population on the basis of FSC and SSC properties. Furthermore, Boolean gating was applied to distinguish cells demonstrating concomitant expression of all selected markers, including: CD134 + CD309 + CD133 + (EPC, endothelial progenitor cells), CD34 + CD144 + (CEC, circulating endothelial cells), and CD34 + CD309 + cells (Supp. Figure 3). All tested cell populations were presented as frequency of specific cell events within all events of leukocytes analyzed, referred as ‘WBC’ – white blood cells.

### Immunoenzymatic Assessment of SDF-1

Isolated plasma samples were used for immunoenzymatic analysis of SDF-1 level in SDF-1-deficient patients and healthy control group. Materials were tested in accordance with instructions included in ELISA DuoSet kit for detection of SDF-1/CXCL12 (R&D Systems). Selected assay allowed for detection of SDF-1 within range of 31.2 – 200 pg/ml. Data were acquired with the use of LEDETECT96 microplate reader (Labexim, Lengau, Austria).

### Statistical Analysis

Biostatistical analysis of the acquired data was performed using GraphPad Prism 9.0.0 statistical software (GraphPad Prism Inc., San Diego, CA, USA). Depending on data distribution obtained results were analyzed with the use of parametric or non-parametric tests for paired or unpaired data. Statistical significance was set at 0.05, with asterisks or p values indicating power on the graphs: *—*p* < 0.05, **—*p* < 0.01, ***—*p* < 0.001, ****—*p* < 0.0001. Violin plots with median values and quartiles were used when IGF-1-deficient and healthy control group were compared. Variations in time were visualized with graphs demonstrating mean change values (and standard deviation) of specific populations frequency compared to time 0 (before therapy). Correlations were performed using non-parametric Spearman test, and presented as correlation coefficients on heat maps, and asterisks distinguishing statistically significant associations. Depending on the r value, obtained results were considered as: weak (0.2 – 0.39), moderate (0.4 – 0.59), strong (0.6 – 0.79) or very strong (0.8 – 1.0) correlations.

## Results

### Peripheral Blood Levels of VSEL and HSC in IGF-1-Deficent Patients

**Fig. 1:**
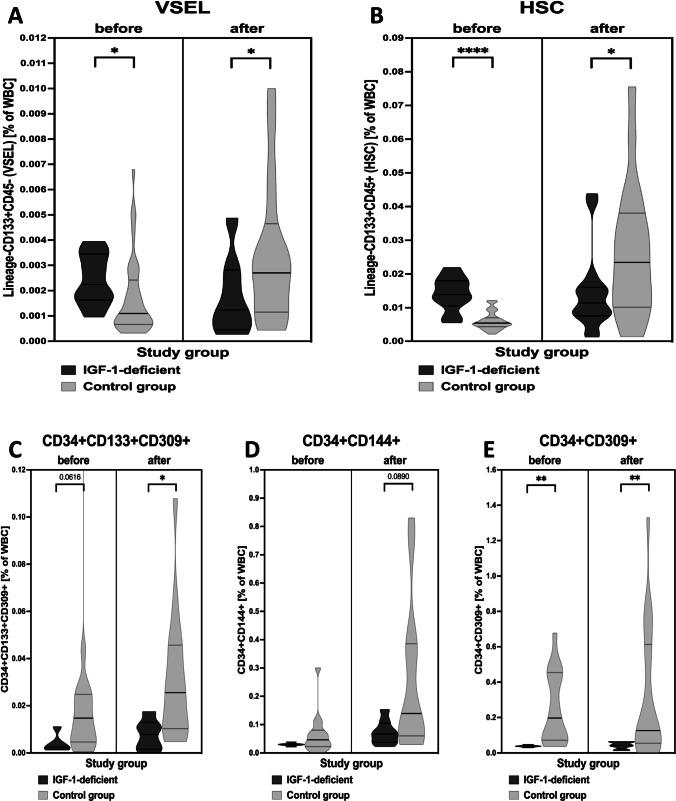
Differences between IGF-1-deficient patients and control group of children in context of VSELs (**A**), HSC (**B**), and endothelial populations of stem cells: EPC (CD34 + CD133 + CD309 +), CEC (CD34 + CD144 +) and CD34 + CD309 + (C-E). Demonstrated data include results obtained before therapy and at 4-5.^th^ year of observation (data presented as violin plot with median and quartiles shown) (asterisks indicate significant p values: *—*p* < 0.05, **—*p* < 0.01, ***—*p* < 0.001, ****—*p* < 0.0001)

First, we evaluated selected stem cells in peripheral blood of IGF-1-deficient patients prior treatment application. Initial analysis revealed significantly higher values for both, VSELs (*p* = 0.0278) and HSCs (*p* < 0.0001) in patients with IGF-1 deficiency compared to control subjects (Fig. 1A-B). Regarding endothelial cells-related events, only CD34 + CD309 + cells (*p* = 0.0019) were significantly reduced in IGF-1-deficient patients, with similar tendency observed in EPC (CD34 + CD309 + CD133 + cells) (*p* = 0.0616) (Fig. 1C-D).

### Peripheral Blood Stem Cells Response to Increlex Application in IGF-1-Deficient Patients

**Fig. 2:**
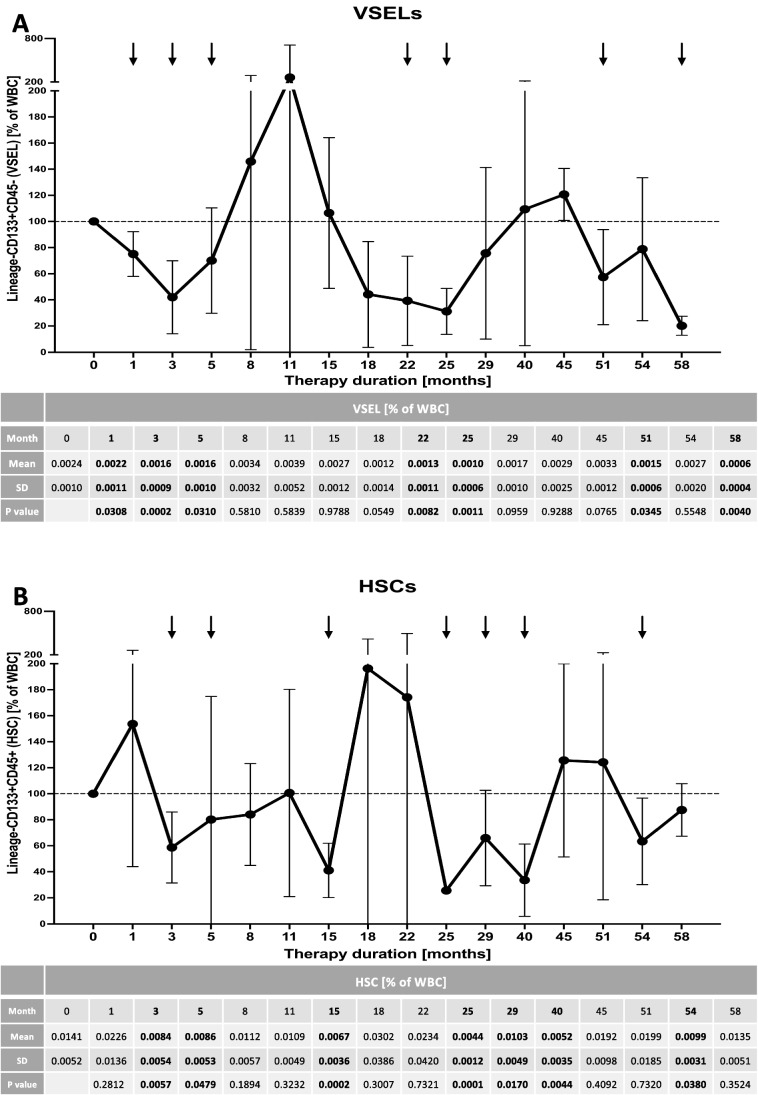
Effects of Increlex application on changes within Very Small Emryonic-Like cells (VSEL; Lineage-CD45-CD133 +) (**A**) and Hematopoietic Stem Cells (HSC; Lineage-CD45 + CD133 +) (**B**) in IGF-1-deficient pediatric patients. Mean change in time of selected parameters was plotted on the graph with standard deviation (SD) included (black vertical arrows indicate statistically significant changes compared to pre-treatment values (Time 0))

**Fig. 3:**
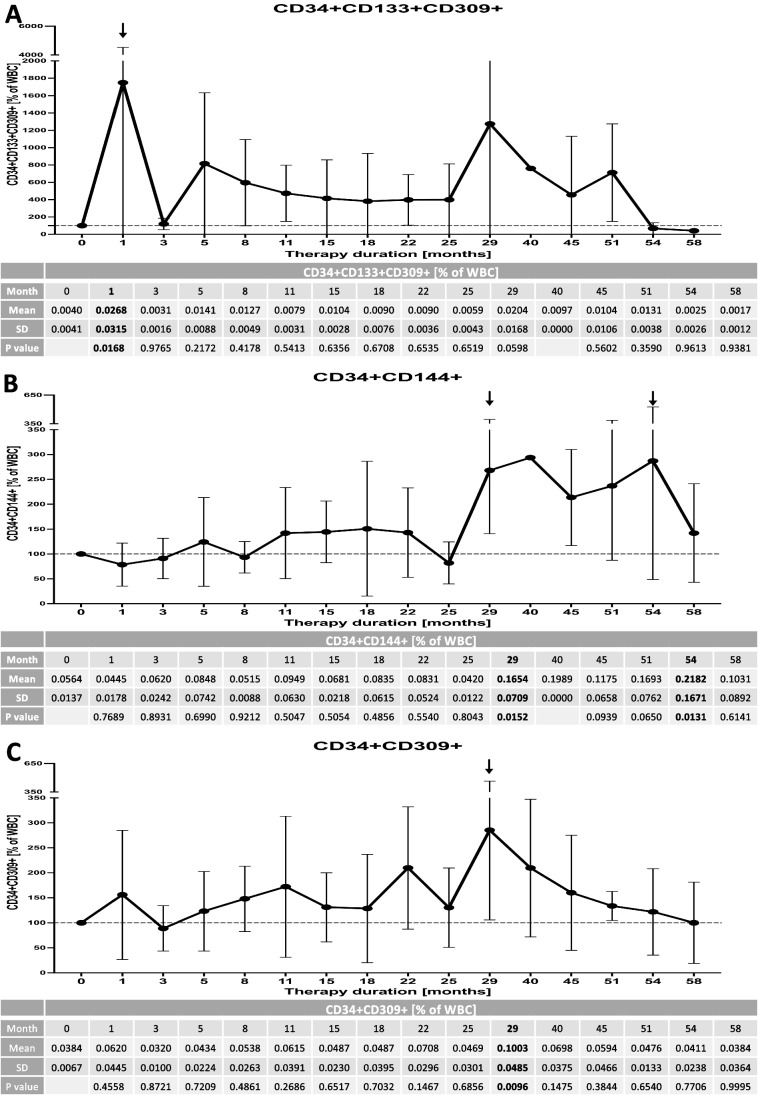
Increlex treatment effects on changes within Endothelial Progenitor Cells (EPC; CD34 + CD133 + CD309 +) (**A**), Circulating Endothelial Cells (CEC; CD34 + CD144 +) (**B**) and CD34 + CD309 + cells (**C**) in IGF-1-deficient pediatric patients. Mean change in time of selected parameters was plotted on the graph with standard deviation (SD) included (black vertical arrows indicate statistically significant changes compared to pre-treatment values (Time 0))

In the course of 4–5 years of therapy we observed essential changes within peripheral population of VSEL and HSC predominantly. Despite periodic elevations of peripheral levels of VSEL and HSC, final effect of Increlex application was associated with decline in these populations of stem cells. In reference to VSELs, significant decrease was observed at first 5 months of therapy, later at 22^nd^ and 25^th^ month, and finally at the end of monitoring at 51^st^ and 58^th^ month (Fig. 2A). These changes were concomitantly followed by a gradual decline in HSC levels. Most significant differences were found after 3–5 months of Increlex use, then at 25^th^ to 40^th^ month, with only slight declines in HSC at 15^th^ and 54^th^ month (Fig. 2B). In contrast to observed alterations in VSELs and HSCs, endothelial stem cells did not demonstrate significant response to the treatment regimen applied. Only transient increase in EPC was found at 1^st^ month of Increlex use, and transiently after 29^th^ month in reference to CD34 + CD144 + and CD34 + CD309 + cells (Fig. 3A-C). At last year of therapy, we tested stem cell levels in reference to their age-matched healthy controls. We found that Increlex application did not only reduce VSELs and HSCs in IGF-1-deficient patients, but also allowed to obtained significantly lower values compared to control group (Fig. 1A-C).

### SDF-1 Concentration in IGF-1-Deficient Patients and Its Response to Increlex Therapy

**Fig. 4:**
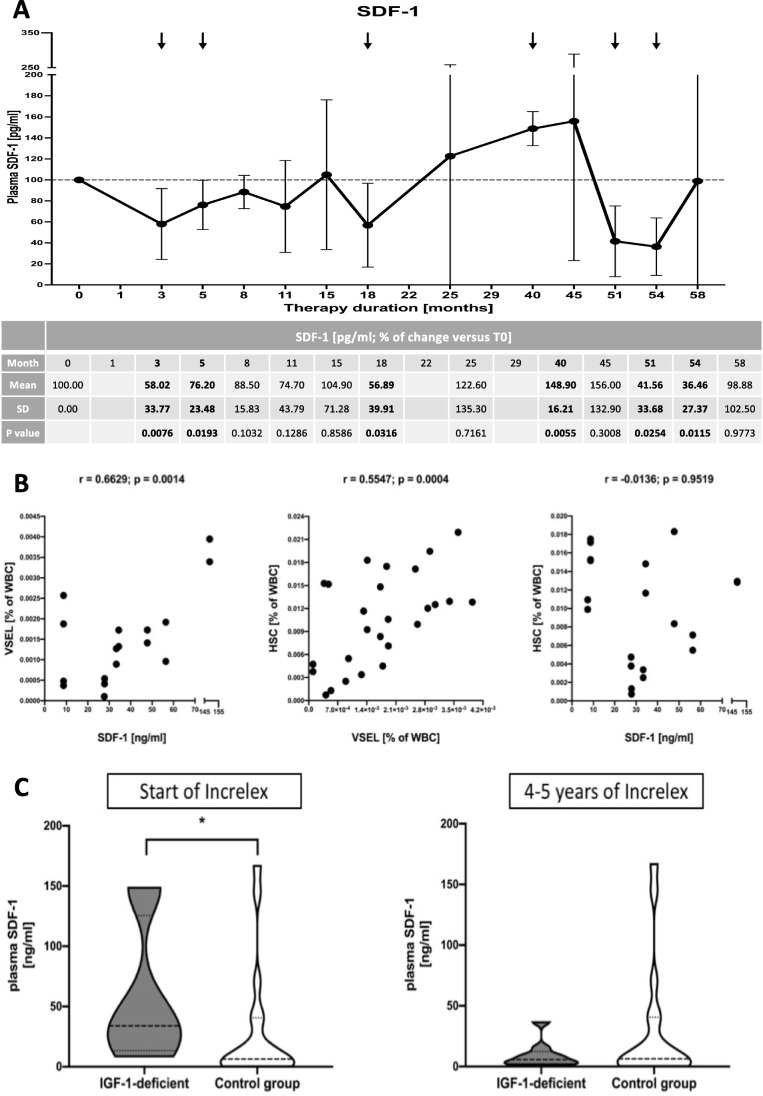
Changes in the SDF-1 plasma concentration in IGF-1-deficient patients subjected to Increlex treatment. Therapy-related alterations in SDF-1 level presented as mean percentage change (**A**) (black vertical arrows indicate statistically significant changes compared to pre-treatment values (Time 0)). Analysis of mutual correlation between SDF-1, VSEL and HSC before Increlex application (**B**). Comparison of SDF-1 values within IGF-1-deficient patients versus healthy control group, at admission and after 4–5 years of Increlex therapy (**C**) (asterisks indicate statistically significant values: *—*p* < 0.05)

Changes of SDF-1 concentration were comparable to variations in VSEL/HSC levels after Increlex application. Thus, decrease in SDF-1 plasma level was initially found at 3-5^th^ month of therapy, and subsequently, at 15^th^ and after 45^th^ month. Lower values of the chemokine seemed to be maintained until the end of patients monitoring (Fig. 4A).

Considering simultaneous decline in both, VSEL, HSC and SDF-1, we intended to evaluate mutual association between these parameters. We found strong correlation of initial plasma SDF-1 levels with frequency of VSELs (r = 0.6629). VSELs also demonstrated strong correlation with HSC values (r = 0.5547). Interestingly, however, no association has been shown between SDF-1 peripheral HSC levels (Fig. 4B).

SDF-1 levels in patients with IGF-1 deficiency were increased before Increlex therapy, thus, complementary to pre-treatment values of VSEL and HSC. Increlex application led to significant changes after 4–5 years of therapy diminishing these differences (Fig. 4C).

### Association Between Tested Cells and Clinical Characteristic of Patients with IGF-1 Deficiency

**Fig. 5:**
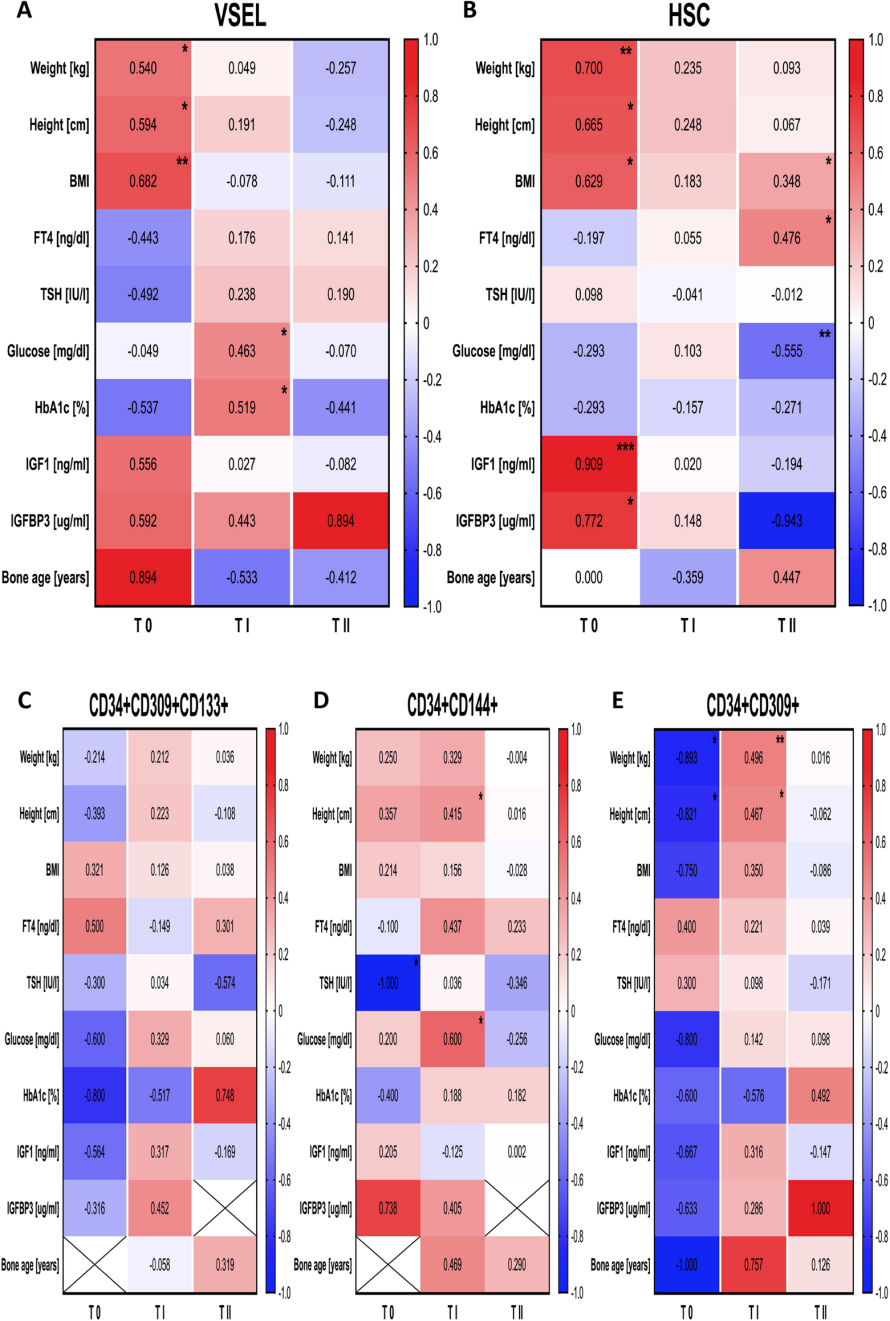
Determination of mutual associations between studied stem cell populations and clinical and laboratory results at three main stages of Increlex therapy: before treatment (T0), 3^rd^-25^th^ (TI) and 29^th^-58^th^ (TII) month of treatment (correlation coefficient values mapped, statistically significant correlations indicated with asterisks: *—*p* < 0.05, **—*p* < 0.01, ***—*p* < 0.001, ****—*p* < 0.0001)

VSEL and HSC were found to strongly correlate with weight, height and BMI of the studied subjects, mostly affected by Increlex application. Furthermore, moderate correlation of VSELs with free thyroxine (fT4) and thyroid stimulating hormone TSH levels were reported. Slight tendency shown for VSELs correlation with IGF-1, IGF-1 binding protein 3 (IGFBP3), and bone age (based on hand and wrist radiograph analysis). Regarding HSCs, similar results were only observed in context of IGF-1 and IGFBP3. At later stages of the therapy links between VSELs and glucose or glycosylated hemoglobin (HbA1c) (at 3^rd^ – 25^th^ month of therapy), and HSC versus BMI and fT4 (positive correlation) or glucose (negative correlation) were reported (at 29^th^ – 58^th^ month of treatment) (Fig. 5A-B).

EPC levels demonstrated tendency for correlation with weight and height, glucose matebolism-related parameters (glucose and HbA1c), and IGF-1 and IGFBP3 levels. Comparable associations were found in reference to CD34 + CD309 + cells. Nevertheless, found correlations were also influenced by Increlex therapy (Fig. 5C-D).

### Effects of Increlex Application on Growth Rate of Studied IGF-1-Deficient Patients

**Fig. 6:**
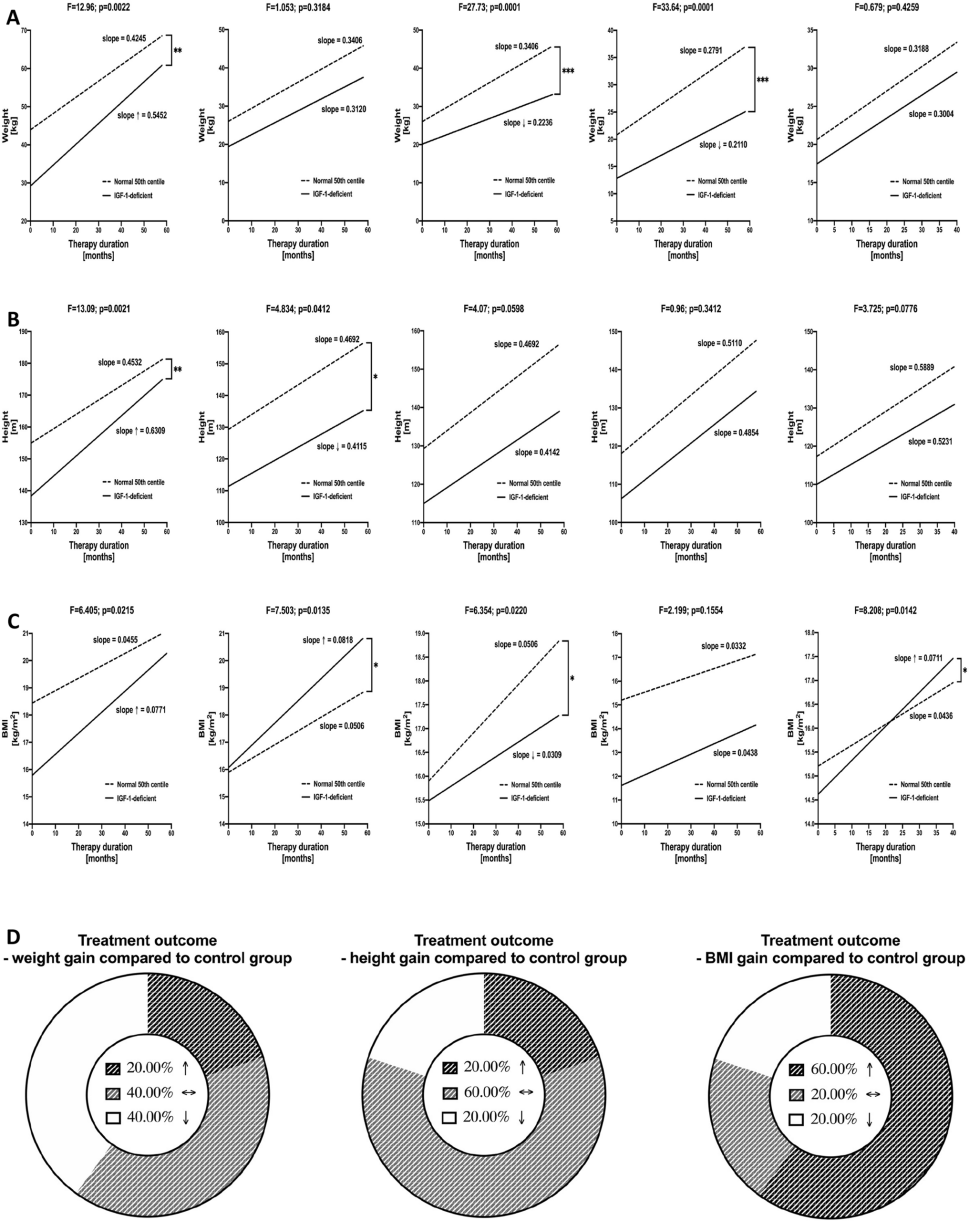
Comparative analysis slopes as visualization of weight, height and BMI differentials between IGF-1-deficient patients and healthy control group. Individual regression lines for each patient with corresponding control group were demonstrated for weight (**A**), height (**B**) and BMI (**C**) increase in time. Data supported by donut graphs with frequency of subjects within groups of higher, lower and unchanged values for the parameters in response to Increlex application (**D**) (slope analysis data presented as regressions of values in time within IGF-1-deficient and proper control group, with slope value) (statistically significant differences indicated with asterisks: *—*p* < 0.05, **—*p* < 0.01, ***—*p* < 0.001, ****—*p* < 0.0001)

Considering influence of Increlex on VSEL and HSC, we monitored growth-related parameters to verify the therapy efficacy on improving development disturbances. Slope analysis of the regression lines was applied to compare intensification of change rates between IGF-1-deficent and healthy patients. 60% of subjects demonstrated at least comparable or even higher rate of growth compared to healthy children. Others were also found to improve their weight in response to Increlex application, however, at slightly lower degree (Fig. 6A, D). For most of patients’ height rates, these values were similar or higher compared to age-matched healthy subjects. Noteworthy, an increase in growth, supported by Increlex, was achieved in all IGF-1-deficient subjects (Fig. 6B, D). We also found that 80% of the Increlex-treated patients achieved at least comparable rate of BMI changes compared control group (Fig. 6C, D).

## Discussion

Significance of IGF-1 axis has been shown to play crucial role in differentiation and growth of cells and tissues [1]. Recently, disturbances in these processes were studied intensively in Laron syndrome patients – suffering from growth disorder but simultaneously demonstrating relatively high longevity [6, 27]. It might be tempting to presume that significantly reduced levels of IGF-1 in IGF-1 deficiency syndrome patients might lead to reduced growth associated with disturbances in stem cells. Since the discovery of VSELs and their presence even in the adult tissues, their use in regeneration has become a study aim for numerous researchers. Although, their limited numbers around body required a lot of effort made by later studies to reveal their potential and possible implications in medicine [10]. To date, presence of VSELs has been confirmed in different tissue compartments including heart, bones, lungs, in numerous cases, detected together with HSCs [11, 12, 20, 28]. Thus, that embryonic-like cell population can be considered an essential element in development and regeneration of all main tissues and organs. With their demonstrated decline with age [11], their significance is most pronounced in the period of intensive growth and maturation.

Here, IGF-1 deficiency syndrome patients exhibit significantly higher frequency peripheral blood VSELs and HSCs. These results are in accordance with previously reported increased ratio of these cells in mice model of growth disturbances. Interestingly, VSELs of these study subjects demonstrated significantly higher level of *Oct4* demethylation – pluripotency regulator, potentially as a response to reduced IGF-1 signaling in declined GH receptor presence [16]. Thus, accumulation of studied stem cells in blood might also be associated with induced expression of genes related to pluripotency. However, question arises why VSEL or HSC high numbers were not able to induce growth of the subjects? That phenomenon could be explained by low IGF-1 level reported in the IGF-deficient patients. Previous studies demonstrated that IGF-1 signaling is important for osteoblasts expansion together with engraftment of HSC [2]. Previous reports indicated role of SDF-1 in regeneration through mobilization of progenitor cells at site of injury [29]. Considering that, decline in peripheral protein level might be associated with higher tissue concentration, thus, leading to increased involvement of VSELs and HSCs in tissues development. Implementation of recombinant IGF-1 in therapy could support proper nesting of HSCs, presumably also VSELs, in the tissues of interest.

In the course of Increlex therapy implementation, we found that populations of HSCs and VSELs are gradually decreasing over time. Considering the fact that HSC were found in the past to be one of the potential source of adipocytes [18], we presume that intensified growth ratio can lead to increased utilization of these cells, possibly also VSEL population. That could be additionally associated with improved activity of the IGF-1 axis and migration of these cells into developing tissues. Decline in both VSEL and HSC populations, presumably as a result of their participation in tissues expansion, can also involve SDF-1 activity. Stromal derived factor 1 (SDF-1), among other factors (including hormones—FSH), has been already demonstrated to be efficient mobilization protein, inducing release of stem cells like VSELs from bone marrow [22]. HSCs have also been reported to be involved by SDF-1 into damaged tissues regeneration, through modulation of progenitor cells within damaged mice hepatic tissue [28]. We speculate that initially high concentrations of plasma chemoattractant SDF-1 might originate from unresponsiveness of monitored stem cells – associated with IGF-1 axis disturbances [30]. Restored levels of IGF-1 in patients were followed by a decrease in peripheral VSEL and HSC, and reduced SDF-1 level. Cumulatively, these data emphasize high probability of our hypothesis of monitored stem cells involvement in tissues development.

Previous studies indicated crucial role of VSELs in the animal model of bone defect regeneration. Studies revealed that bone marrow-derived mononuclear cells were significantly less efficient in regeneration when deprived of VSELs. Noteworthy, presence of VSELs was associated with reduced inflammation, based on CD68 + macrophages activity, and lower levels of pro-inflammatory cytokines: IL-1beta and MCP-1 [20]. Those studies could be partially linked to phenomenon present in children growth and associated increase in size and width of the bones. We are aware of the limited number of subjects where correlations between bone age and studied stem cells has been tested. However, we provide basis to consider VSELs a crucial participant in the tissue growth. Furthermore, changes in VSELs were followed by high efficacy of Increlex treatment as height and BMI change rate was higher or comparable to healthy controls in 80% of cases. Such beneficial effects of the therapy are in accordance with data from Israel team where recombinant IGF-1 application significantly improved height of the Laron subjects [31]. Although the up-to-date data questions efficacy of the rhIGF-1 application [8], here we shown that most of the patients achieved at least comparable growth versus healthy subjects. Importantly, we demonstrated not only significantly improved growth-related values [9], but also change rate of these parameters. Regarding positive correlations between HSCs and anthropometric data, those stem cells seemed to have as important role in growth as VSELs.

In reference to HSC-related phenomena prior and after therapy, we found significant correlation of that population with IGF binding protein 3 (IGFBP3) – responsible for IGF-1 distribution and activity [3]. These data might support previously reported role of IGFBP2 in promoting survival and circulation of HSCs. Despite indication of mechanism independent from IGF-1 signaling [32], here, we demonstrated strong link between IGF-1 levels and HSCs. Taking into account described importance of IGF-1 in HSC mobilization [2], we presume that normal level of the hormone together with IGFBP3 is essential in stem cells proper distribution in growth. Previously, IGFBP3 protein was found to be moderately associated with growth velocity, suggesting its role as predictor of response to recombinant GH therapy [33]. These findings are substantially supported by our data, additionally extended by knowledge of tested VSELs and HSCs strong association with both IGFBP3 and height.

In context of CD34+ cells, only pre-treatment CD34+CD309+ cells were found to have lower levels in IGF-1-deficient patients. Despite an increase of CD34+ cells in response to GH replacement therapy was reported previously [34], here, we showed no change in EPC, CEC or CD34+CD309+ cells. Additionally, we found that CD34+ cells expressing VEGF receptor (CD309) were found to initially correlate negatively with heigh and weight of the patients. Essential role of these cells in context of vasculo- and angiogenesis cannot be excluded, however, in reference to growth those populations does not seem to play significant role.

Cumulatively, our data provide clinical evidence of critical role of IGF-1 signaling pathway in growth of pediatric patients with IGF-1 deficiency syndrome. Furthermore, we found essential importance of IGF-1 in possible mobilization of VSELs and HSCs into developing tissues. Thus, implementation of rhIGF-1—Increlex, could be more precisely associated with supporting efficient nesting of developing tissues. We must always remember about possible complications associated with intensified activity of stem cells in response to growth factors, including promotion of tumor cells [18]. Therefore, despite favorable effects reported here, regular monitoring of the Increlex therapy is required [35]. Regarding study limitations, we must note extremely low incidence of IGF-1 deficiency syndrome (estimated to be around 500 worldwide for Laron syndrome) [36]. Therefore, relatively small number of our studied group, consisting of all the province patients, seems to be justified. However, we hope that further multi-center studies would be possible to support our conclusions, complemented with mechanistic explanation of risen hypothesis of VSEL/HSC role in growth. Despite limitations, current data shed a significant light on VSELs and HSCs participation in growth-related phenomenon in the course of IGF-1 deficiency. In addition, here we provide important basis for further research on stem cells and their role in clinical aspects associated with development disturbances and related therapeutic approaches.

## Supplementary Information

Below is the link to the electronic supplementary material.

Supplementary file1 (JPG 2.48 MB)

Supplementary file2 (JPG 397 KB)

Supplementary file3 (JPG 835 KB)

## Data Availability

The study data are available upon request and agreement of the corresponding authors.
