# Supplementary figures and images for: Alterations in Stem Cell Populations in IGF-1 Deficient Pediatric Patients Subjected to Mecasermin (Increlex) Treatment
Source: Stem Cell Rev Rep. 2022 Oct 21;19(2):392–405. doi: 10.1007/s12015-022-10457-2 (PMC9902328; doi:10.1007/s12015-022-10457-2)

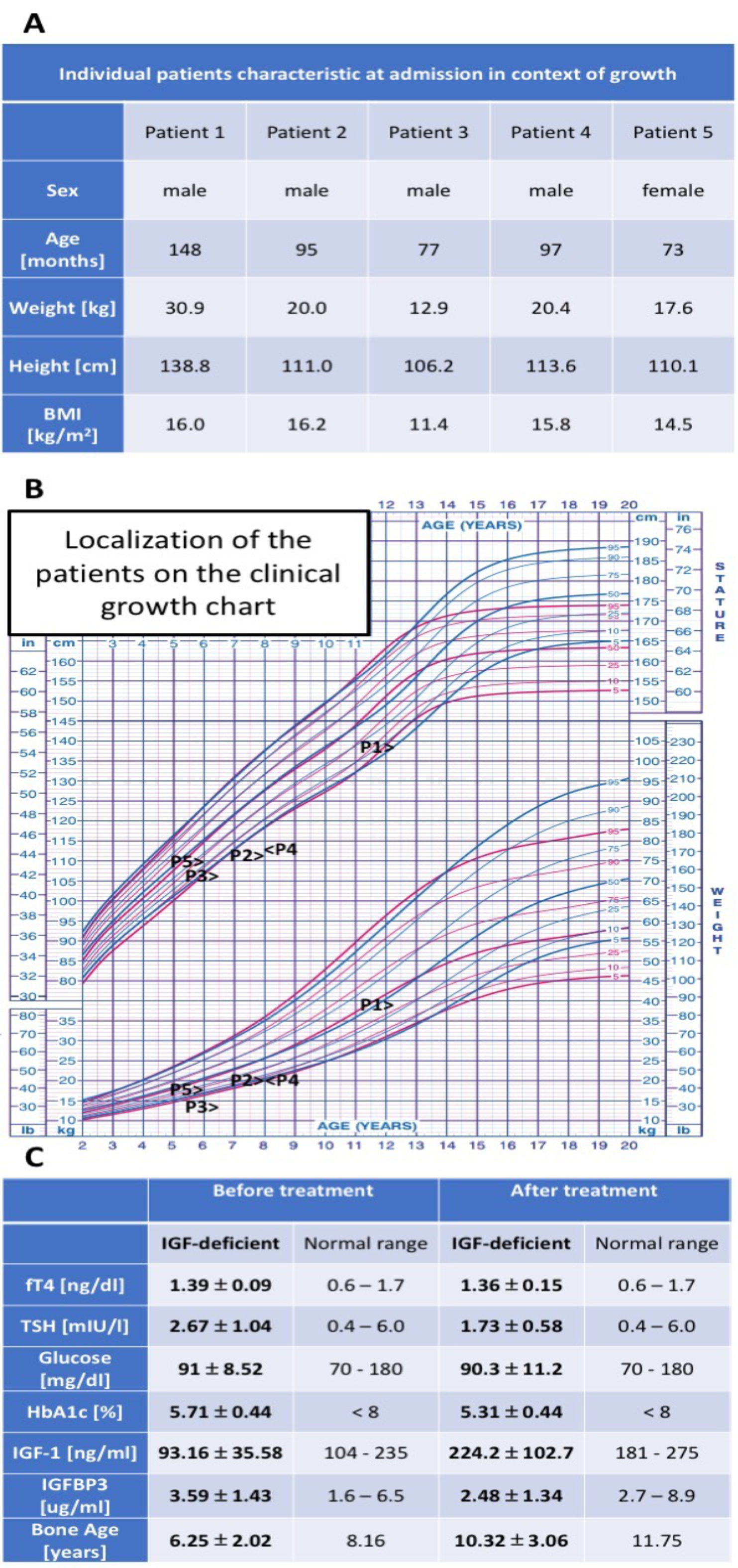

Supplement: Supplementary file 1 — Supplementary file1 (JPG 2.48 MB) [file 12015_2022_10457_MOESM1_ESM.jpg]

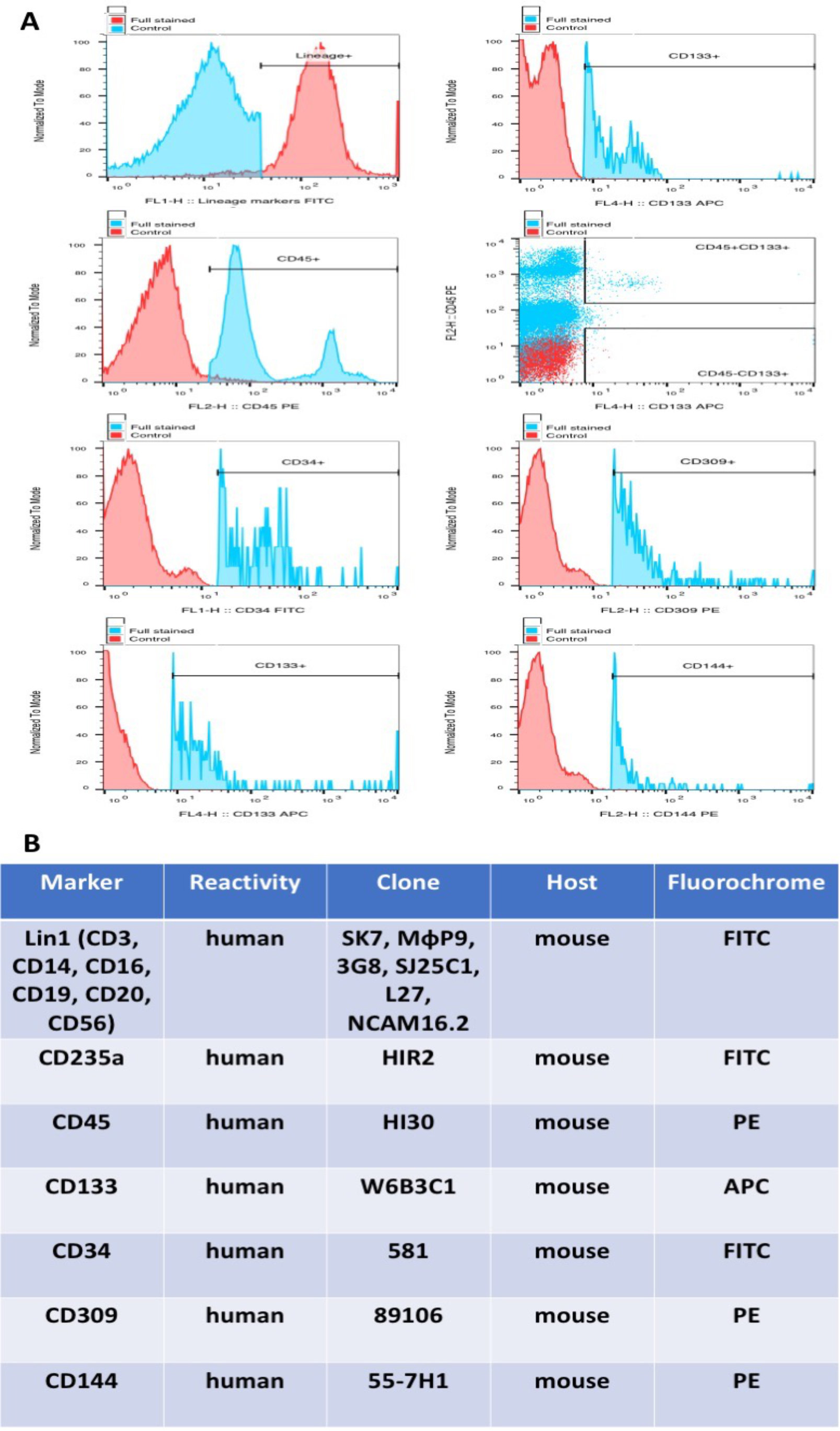

Supplement: Supplementary file 2 — Supplementary file2 (JPG 397 KB) [file 12015_2022_10457_MOESM2_ESM.jpg]

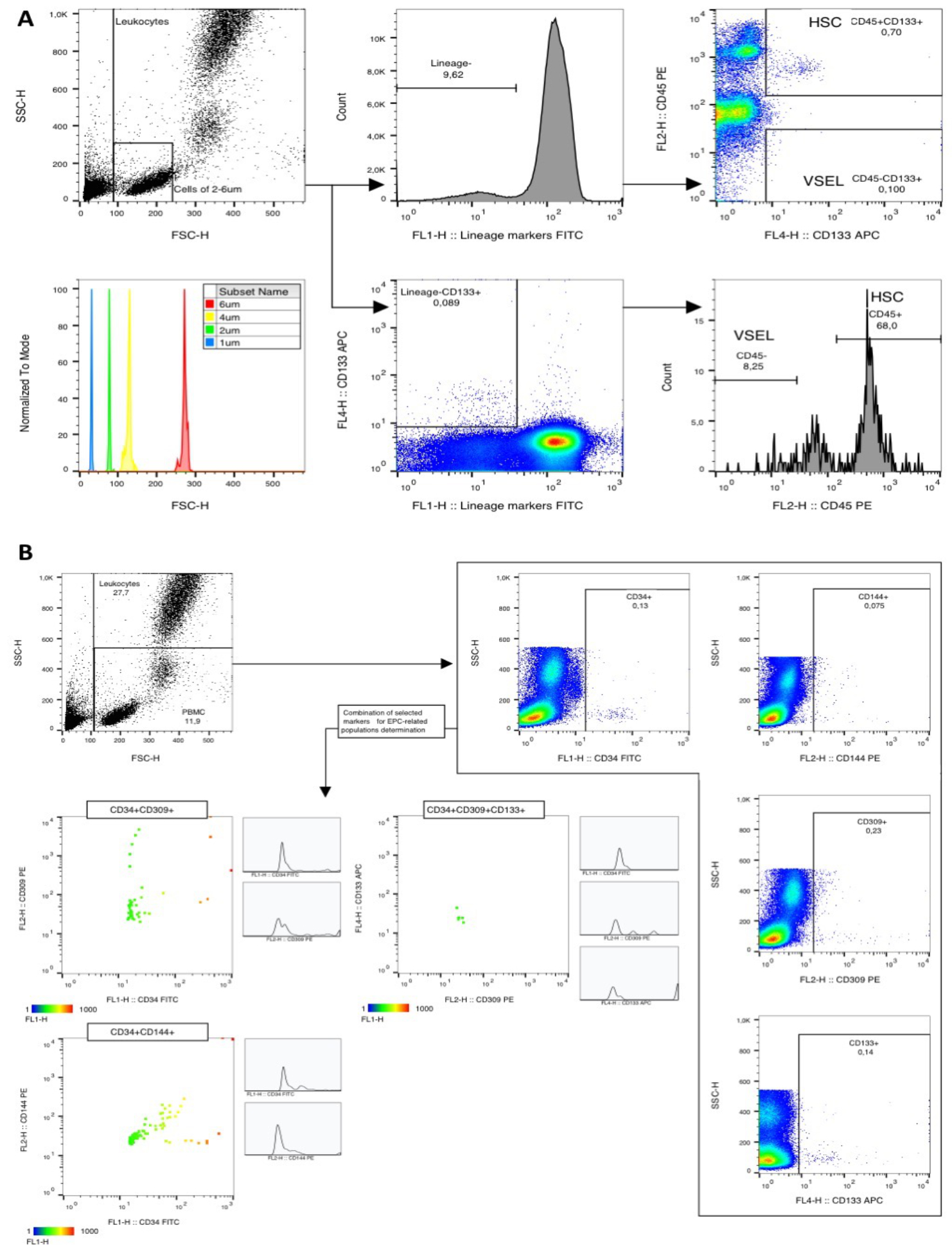

Supplement: Supplementary file 3 — Supplementary file3 (JPG 835 KB) [file 12015_2022_10457_MOESM3_ESM.jpg]
